# GWAS and multi-omics study reveal *OsJAR2* associated jasmonate biosynthesis contributes to Southern rice black-streaked dwarf virus resistance in rice

**DOI:** 10.1186/s12864-025-12159-8

**Published:** 2025-10-29

**Authors:** Shuai Nie, Haiyong Gu, Zhanbiao Li, Lian Zhou, Lixian Cui, Runfeng Wang, Qi Liu, Bixia Qin, Jiansong Chen, Junliang Zhao

**Affiliations:** 1 Rice Research Institute, Guangdong Academy of Agricultural Sciences/Guangdong Key Laboratory of Rice Science and Technology/Guangdong Rice Engineering Laboratory/Key Laboratory of Genetics and Breeding of High Quality Rice in Southern China (Co-construction by Ministry and Province), Ministry of Agriculture and Rural Affairs, Guangzhou, 510640 China; 2 Plant Protection Research Institute, Guangxi Academy of Agricultural Sciences/Guangxi Key Laboratory of Biology for Crop Diseases and Insect Pests/Key Laboratory of Green Prevention and Control on Fruits and Vegetables in South China Ministry of Agriculture and Rural Affairs, Nanning, 530007 China; 3 Guangdong Provincial Key Laboratory of Crop Genetic Improvement, Crops Research Institute, Guangdong Academy of Agricultural Sciences, Guangzhou, China

**Keywords:** Rice (*Oryza sativa*), Southern rice black-streaked dwarf virus (SRBSDV), Comparative transcriptome, Jasmonic acid, *OsJAR2*

## Abstract

**Background:**

Southern rice black-streaked dwarf virus (SRBSDV), transmitted by the white-backed planthopper (WBPH), causes severe yield losses in rice across Asia. However, elite resistant germplasms and molecular defense mechanisms remain elusive, hindering breeding efforts.

**Results:**

Screening of 195 international diverse rice accessions identified a highly resistant (up to 0% disease incidence) variety (R91), exhibiting dual resistance to SRBSDV and rice black-streaked dwarf virus (RBSDV). Multi-omics analysis revealed a rapid defense activation in R91, with an increase in Jasmonic Acid (JA) and Jasmonic Acid-Isoleucine (JA-Ile) by 5 days post-inoculation (dpi), along with upregulation of more than 2,000 defense genes. In contract, susceptible line showed declining JA and JA-Ile level along with suppressed defense responses. Time-ordered co-expression networks pinpointed that *OsJAR2* (encoding a JA-Ile synthase, *LOC_Os01g12160*) may act as a hub of resistance molecular network. Genome wide association study (GWAS) identified a novel SRBSDV resistance quantitative trait locus (*qSRBSDV1-1*) co-localizing with *OsJAR2*, and haplotype analysis validated *OsJAR2* as the candidate causal resistance gene, further providing genetic evidence for its role in SRBSDV defense.

**Conclusions:**

Our study identifies a highly SRBSDV-resistant rice germplasm, offering valuable genetic resource for both resistance research and breeding programs. We demonstrate that rapid JA biosynthesis activation and coordinated defense gene expression form the molecular basis of resistance in this accession. Crucially, we pinpoint *OsJAR2* as a novel functional resistance gene, with its associated resistant haplotype serving as a robust molecular marker for marker-assisted selection (MAS). These findings advance our understanding of SRBSDV resistance mechanisms and provide a genetic toolkit for developing elite, disease-resistant rice varieties.

**Supplementary Information:**

The online version contains supplementary material available at 10.1186/s12864-025-12159-8.

## Background

Southern rice black-streaked dwarf virus (SRBSDV) is transmitted by the white-backed planthopper (WBPH, *Sogatella furcifera*) in a persistent, circulative, and propagative manner [1–4]. This virus has emerged as a devastating pathogen, causing substantial yield losses of up to 70% in severely infected rice fields, particularly in Southern China, Southeast Asia, and even extending to regions as far north as Japan [1–4]. As a double-stranded RNA virus belonging to the genus *Fijivirus* in the family Reoviridae, SRBSDV shares similarities with rice black-streaked dwarf virus (RBSDV) [5], and was first identified in Guangdong province, China [6].

The implications of SRBSDV are immense, not only compromising rice quality but also posing a serious threat to agricultural economies reliant on rice cultivation as a staple crop. Despite current management strategies, such as the application of pesticides and crop rotation, achieving limited success in controlling SRBSDV outbreaks, breeding for resistant rice varieties has emerged as a highly economical, environmentally sustainable, and viable long-term strategy to combat this virus. Recent studies have identified rice accessions with natural resistance to SRBSDV, underscoring the potential for developing resilient cultivars [7, 8]. Understanding the mechanisms underlying this resistance is crucial for advancing rice breeding programs aimed at enhancing viral resistance.

The advancement of multi-omics techniques has significantly broadened our understanding of the fundamental genes and molecular pathways involved in a plant’s response to pathogens, as well as the development of resistance mechanisms [9]. Specifically, transcriptome analysis has been instrumental in providing invaluable insights into the comprehensive gene expression responses of rice to biotic stress, emphasizing its role in enhancing resistance. Furthermore, the adoption of Time-Ordered Gene Co-Expression Networks (TOGCNs) has emerged as a powerful tool for dissecting time-series transcriptome data, revealing dynamic alterations in gene expression and their transitions across various biological processes [10]. This innovative method transcends the limitations of traditional co-expression network analysis, enabling the exploration of temporal dynamics and the investigation of gene regulatory mechanisms under diverse developmental stages and environmental conditions. As a result, TOGCNs harbor significant potential for unraveling gene regulatory mechanisms under temporal dynamics, subsequently identifying central genes and pivotal pathways involved in combating SRBSDV and offering crucial insights into the molecular mechanisms of resistance. However, there are only a few comprehensive studies that aim to elucidate the molecular mechanisms, and dissect the molecular network related to rice response and resistance to SRBSDV, particularly utilizing network analysis methods like TOGCNs [11].

In this study, we evaluated SRBSDV resistance in RPD2, an international rice diversity panel [12], through field trials and artificial inoculations. Our investigation identified R91 as a highly resistant accession. Notably, R91 was previously recognized for its resistance to RBSDV in our earlier work [7]. Using R91 as a model, we employed integrated approaches—including hormone profiling, time-course transcriptomics, TOGCN, and genetic analysis—to dissect the resistance mechanism. Our analysis revealed that rapid activation of jasmonic acid (JA) biosynthesis may play a pivotal role in SRBSDV resistance, with *OsJAR2* emerging as a possible functional gene regulating SRBSDV resistance in rice.

Our study identified a valuable SRBSDV-resistant germplasm and revealed the crucial role of rapid transcriptional responses and JA signaling in resistance. We further implicated *OsJAR2* as a likely functional hub gene in the JA-mediated resistance network, offering a promising target for breeding resistant rice and advancing the understanding of viral resistance mechanisms.

## Materials and methods

### Plant materials

A total of 195 rice accessions were obtained from RPD2, an international rice diversity panel exhibiting extensive genetic diversity and wide geographic distribution [12]. We assessed the resistance to SRBSDV using these accessions, which originated from 34 countries and spanning 13 regions (Table S1). Both field trials and artificial inoculation tests were conducted to evaluate their resistance levels. We ultimately identified a highly resistant cultivar, accession number 63 (designated as R91 in this study), as well as a susceptible variety, accession number 41 (designated as S1 in this study), for further analysis. All SRBSDV-infected plant materials and subsequent cultivation took place in Nanning, Guangxi province. The plants were grown in a greenhouse maintained at 28–30 °C with a 12-hour light/dark cycle.

### Evaluation of disease resistance using artificial inoculation

Rice plants exhibiting typical symptoms, such as dark green and dwarf, were collected from a field in Nanning city, Guangxi Province and confirmed by RT-PCR with SRBSDV-specific primers [13]. Those testing positive for SRBSDV were then cultivated in the insect-proof greenhouse for artificial inoculation and further identification.

WBPH were also collected from a rice field in Nanning city, Guangxi province and reared on healthy rice plants until new nymphs emerged. Newly hatched nymphs were then transferred and propagated on the SRBSDV-infected rice plants for more than 14 days to be viruliferous. The proportion of viruliferous WBPH was assessed by RT-PCR with SRBSDV-specific primers. The viruliferous WBPHs were used to inoculate SRBSDV to rice seedlings at three-leaf stage.

Rice seedlings were grown in the insect greenhouse till three-leaf stage, the viruliferous WBPHs were then transferred to the seedlings and kept for 48 h. After inoculation, the seedlings were transplanted in the insect greenhouse for observation of the symptoms and resistant evaluation. Disease incidence (DI) was calculated according to the technical specification for identification and evaluation of rice variety resistance against SRBSDV (NY/T 4455 − 2025).

### Evaluation of disease resistance in field trial

Field trial was conducted in Xingʹan county, Guilin city, Guangxi province. Xing’an county is an important migration route for the WBPHs, and the incidence of SRBSDV was always high. All the 195 rice accessions were randomly planted in the paddy field; each accession had three replicates. To ensure the test effect, TN1 as the susceptible control was evenly distributed among these accessions, and rice seedlings were cultivated in a field setting surrounded by a rice crop affected by SRBSDV. At least 50 seedlings of each accession were grown in the field for natural infection, with a row spacing of 15 cm * 18 cm, each material was planted with 5 rows * 10 plants. Disease incidence (DI) was recorded two months post-transplanting, following standard management practices without the application of pesticides or antivirals. The identification result was deemed valid when the DI of the TN1 exceeded 30%.

### Sample preparation for hormones quantification and RNA sequencing

Samples used for hormones quantification and RNA sequencing comprised four groups: R91+ (resistant line treated with viruliferous insects), R91 (resistant line treated with virus-free insects), S1+ (susceptible line treated with viruliferous insects), and S1 (susceptible line treated with virus-free insects). All plants were artificially inoculated with SRBSDV using the aforementioned disease resistance evaluation method. Samples were collected from each group at 5-, 11-, and 20-days post-inoculation (dpi), with three independent replicates at each time point. Leaf tissues from all 36 individuals, covering both virus-infected and virus-free plants, were harvested for plant hormones quantification, RNA extraction and subsequent transcriptome analysis.

### Plant hormones quantification

Hormone quantification was performed by LC-ESI-MS/MS (UHPLC-Qtrap) at Shanghai Meiji Biology (China) using the following protocol: (i) Standard preparation: Forty-one phytohormone standards were dissolved in methanol to prepare stock solutions, which were then diluted with 50% methanol to generate 10-point calibration curves. (ii) Sample extraction: Frozen rice leaves (100 mg) were homogenized in 498 µL of 80% methanol containing 2 µL SA-D4 internal standard (2 µg/mL), followed by cryogenic grinding (3 min) and ultrasonic extraction (1 h, 4 °C). After adding 25 mg modified salts, samples were vortexed (10 min), centrifuged (12,000 × g, 10 °C, 10 min), and the supernatant (100 µL) was diluted with 60 µL water. (iii) LC-MS/MS analysis: Separations were performed on a Waters BEH C18 column (2.1 × 100 mm, 1.7 μm) using 2 mM ammonium formate with 0.05% formic acid in water/methanol as mobile phases. (iv) Data Analysis: Quantitation was performed using software Sciex (default parameters) with manual validation. Calibration curves were constructed by plotting analyte peak areas against known concentrations (linear regression). Sample concentrations were calculated by substituting the measured peak areas into their respective standard curves and are expressed as nanograms per milligram of fresh weight (ng/mg FW).

### RNA sequencing

Sampled leaves were immediately flash-frozen in liquid nitrogen and kept at −80 °C. Using the NEBNext Poly(A) mRNA Magnetic Isolation Module, mRNA was isolated. RNA quality was assessed using the Agilent 2100 BioAnalyzer. In total, 36 sequencing libraries were constructed using the NEBNext Ultra RNA Library Prep Kit for Illumina. 150 bp paired-end PCR-free libraries were prepared using the NEBNext Ultra II RNA Library Prep Kit for sequencing with an Illumina HiSeq X Ten platform.

### RNA-seq analysis

Short reads were processed with fastp to remove adapter sequences, leading and trailing bases with a quality score below 20, and reads with an average per base quality of 20 over a 4-bp sliding window [14]. The Nipponbare genome (IRGSP-1.0) [15] was used as a reference for reads mapping with HiSat2 (https://daehwankimlab.github.io/hisat2/). Only uniquely mapped paired-end reads were retained for read counting of the genes by featureCounts to generate the count and Transcripts per Kilobase Million (TPM) Table [16]. A comprehensive analysis was conducted on a set of 36 samples, yielding a total of 1,439.31 million high-quality clean reads, corresponding to 215.9 G paired bases (Table S2). On average, 83.21% of these paired-end reads were uniquely mapped to the reference genome, ensuring a reliable dataset for subsequent analyses (Table S2).

Differential gene expression analysis was performed with DEseq2 [17]. And the differentially expressed genes (DEGs) were identified according to the criteria of adjusted *p* value < 0.05 and an absolute log2 fold change (|LFC|) > 2.

### Gene functional annotation

All gene structural annotations are based on release 7 of the MSU Rice Genome Annotation Project [15]. We downloaded the transcription factors list from PlantRegMap, obtaining a total of 2,408 TFs which were classified into 56 families [18]. GFAP was used for de novo gene functional annotation with the plant-specific database [19]. Given the importance of JA metabolism in resistance to SRBSDV, we annotated genes related to the JA biosynthetic and metabolic pathway by gathering enzymatic family annotations from previous studies (Table S3).

### Reconstructing the time-ordered gene co-expression networks

We firstly preprocessed the gene expression data as follows: (i) For each time point, three replicates were treated as one data point. After performing pairwise comparison among different time points within the four treatments (R91, R91+, S1, and S1+), all identified significantly DEGs were combined, resulting in a total of 12,652 significant DEGs. (ii) We calculated the mean TPM for gene expression within each time point group, retaining genes with a mean greater than 1 in any group. (iii) Based on the criteria from the previous two steps, we calculated the median absolute deviation (MAD) of the TPM values for each gene, retaining the top 5,000 genes ranked by descending MAD.

The base R function “cor” was used to calculate the gene co-expression Pearson correlation coefficients (r) between pairs of genes under four different temporal treatments. TOGCN was performed for generating the suggested r cutoff with 0.88 and − 0.65 [10]. The value of r between − 1 and − 0.65 indicates a significantly negative correlation between two genes, while r between 0.88 and 1 indicates a significantly positive correlation. Each gene acts as a node, while a pair of significantly correlated co-expressed genes forms a gene pair that serves as the edge connecting these nodes. The interplay of nodes and edges together constructs a gene co-expression network (GCN). Comparing different GCNs essentially involves identifying gene pairs that are specific to each network. We constructed four independent GCNs based on four sets of time-series gene expression data (R91, R91+, S1, S1+). Significantly positively correlated gene pairs unique to the R91 + GCN were combined to create the R91 + specific GCN, while those specific to the S1 + GCN were merged to form the S1 + specific GCN.

Breadth-first search algorithm [20] was used for GCN clustering with custom Python script. To cluster GCN, it is necessary to select appropriate genes as seeds, whose expression trends should gradually decrease from the first time point to the last time point. We identified 13 optimal genes as seeds using MFSelector (Table S4) [21]. After clustering, the R91 + and S1 + specific GCNs formed Resistant (R91+)- and Susceptible (S1+)- specific time-ordered gene co-expression network (TOGCN), respectively. Ultimately, TOGCNs were visualized in graphs using Cytoscape [22]. The centrality of nodes (gene) in the TOGCN was evaluated using the R package igraph.

### Gene functional enrichment

Hypergeometric tests were performed to determine whether specific functional categories were significantly overrepresented in gene sets. GO functional enrichment was tested using the R package clusterProfiler [23].

### Genome-wide association study

Genome-wide association study (GWAS) was conducted using our previous sequencing data [24], and Gapit version 2 with a mixed linear model (MLM) incorporating kinship matrix and with PCs set to 3 to control for population structure (Fig. S1) [25]. Manhattan plots were generated using CMplot package in R v4.2.2 software (https://github.com/YinLiLin/R-CMplot). The significance threshold was set as *P*-value ≤ 1 × 10^−5^.

### Data analysis and image production

Statistical differences were evaluated using the t-test. Principal component analysis (PCA) and Pearson correlation analyses were performed with built-in functions in the R software environment. The frequency distribution of haplotypes across various global regions was calculated and visualized using the R package geneHapR (https://github.com/ZhangRenL/geneHapR).

## Results

### Screening of SRBSDV resistant accessions using international rice diverse panel

**Fig. 1:**
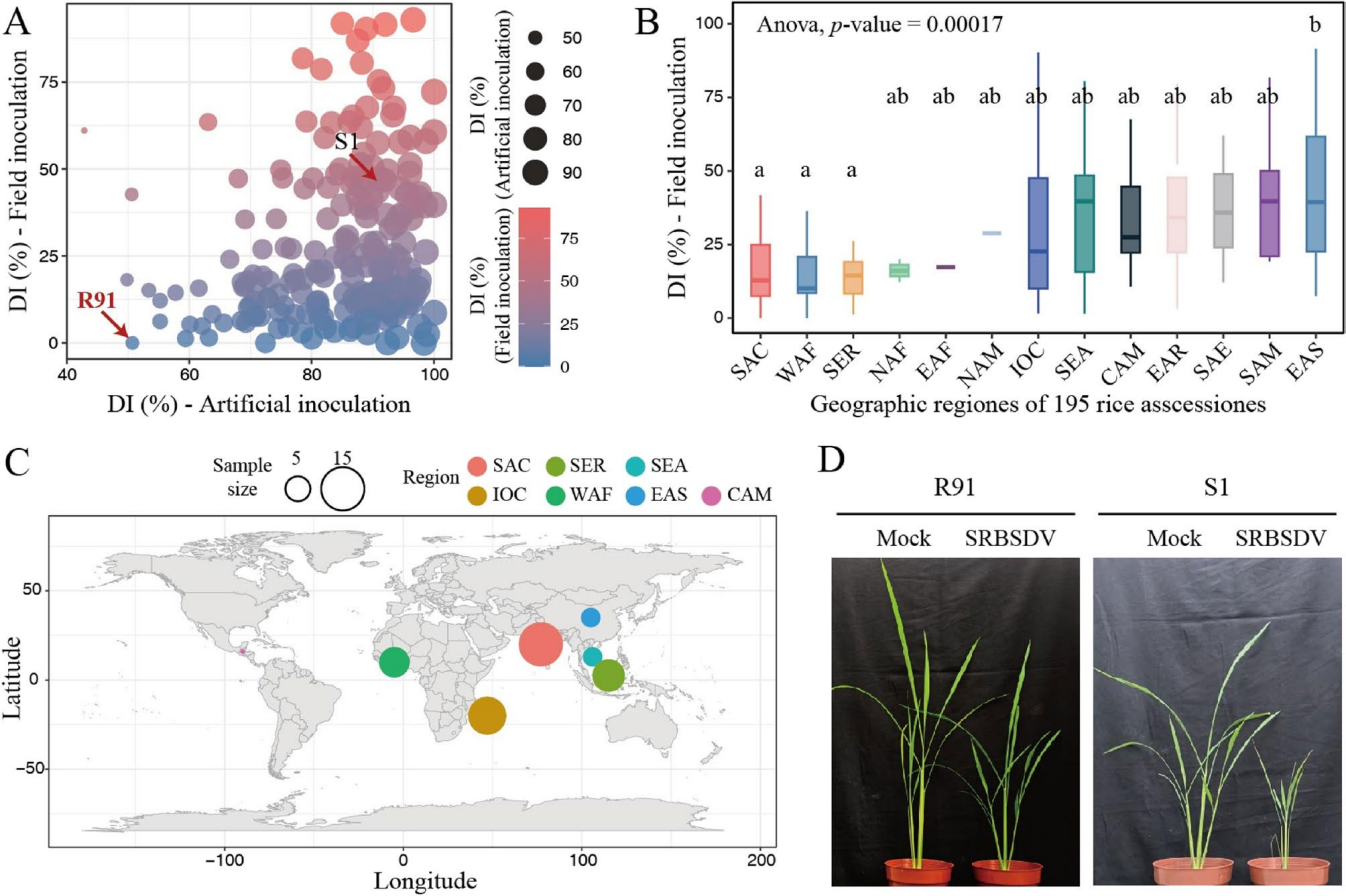
R91 is a stable resistant accession for SRBSDV. (**A**) Disease incidence (DI) of 195 rice accessions under field trial and artificial inoculation. R91 (resistant) and S1 (susceptible) accessions used in subsequent analyses are labeled (arrows). (**B**) The distribution of DI among rice accessions from different geographical regions. CAM, Central America and Caribbean; EAF, East Africa; EAS, East Asia; IOC, Indian Ocean Commission; NAF, North Africa; NAM, North America; SAC, South Asia Central; SAE, South Asia East; SAM, South America; SEA, Southeast Asia; SER, Sea islands; WAF, West Africa. (**C**) Geographic distribution and size of the top 50 accessions with the lowest DI based on field inoculation. (**D**) Representative Mock- (SRBSDV-free WBPH) or SRBSDV-inoculated R91 and S1 plants were photographed at 30 days post inoculation (dpi)

To evaluate SRBSDV resistance and characterize its distribution among different rice varieties, we leveraged a diverse panel composed of 195 rice accessions originating from 34 countries worldwide and spanning 13 regions. All accessions underwent rigorous disease resistance assessment via both field trials and controlled artificial inoculation (Table S1). Considerable variation in DI was observed in the panel, with field test DI ranging from 0% to 92.9% and artificial inoculation DI varying from 42.8% to 100% (Fig. 1A and Table S1).

Analysis of the geographic distribution of resistant accessions showed that accessions from East Asia (EAS; including China and Japan) exhibited significantly higher DI (Fig. 1B). In contrast, accessions from three regions demonstrated significantly lower DI characteristics, including South Asia Central (SAC; including India, Bangladesh, and Nepal), West Africa (WAF; including Burkina Faso, Côte d’Ivoire, Ghana, Guinea, Liberia, Mali, Nigeria, and Senegal), and the Sea Islands (SER; comprising Indonesia, Malaysia, and the Philippines) (Fig. 1B). Among the 50 accessions with the lowest DI in field tests, 15 accessions originated from South Asia Central (Fig. 1C). We identified an *indica* rice variety, R91, from India, exhibiting the constant resistance in both field trial and artificial inoculation (Fig. 1D). Interestingly, this accession has also been identified as a stable and highly resistant resource to RBSDV, a closely related virus to SRBSDV, in our previous study, from which we identified a functional resistance gene for RBSDV [7].

### Resistant accession R91 exhibits quick JAs accumulation post-SRBSDV inoculation

**Fig. 2:**
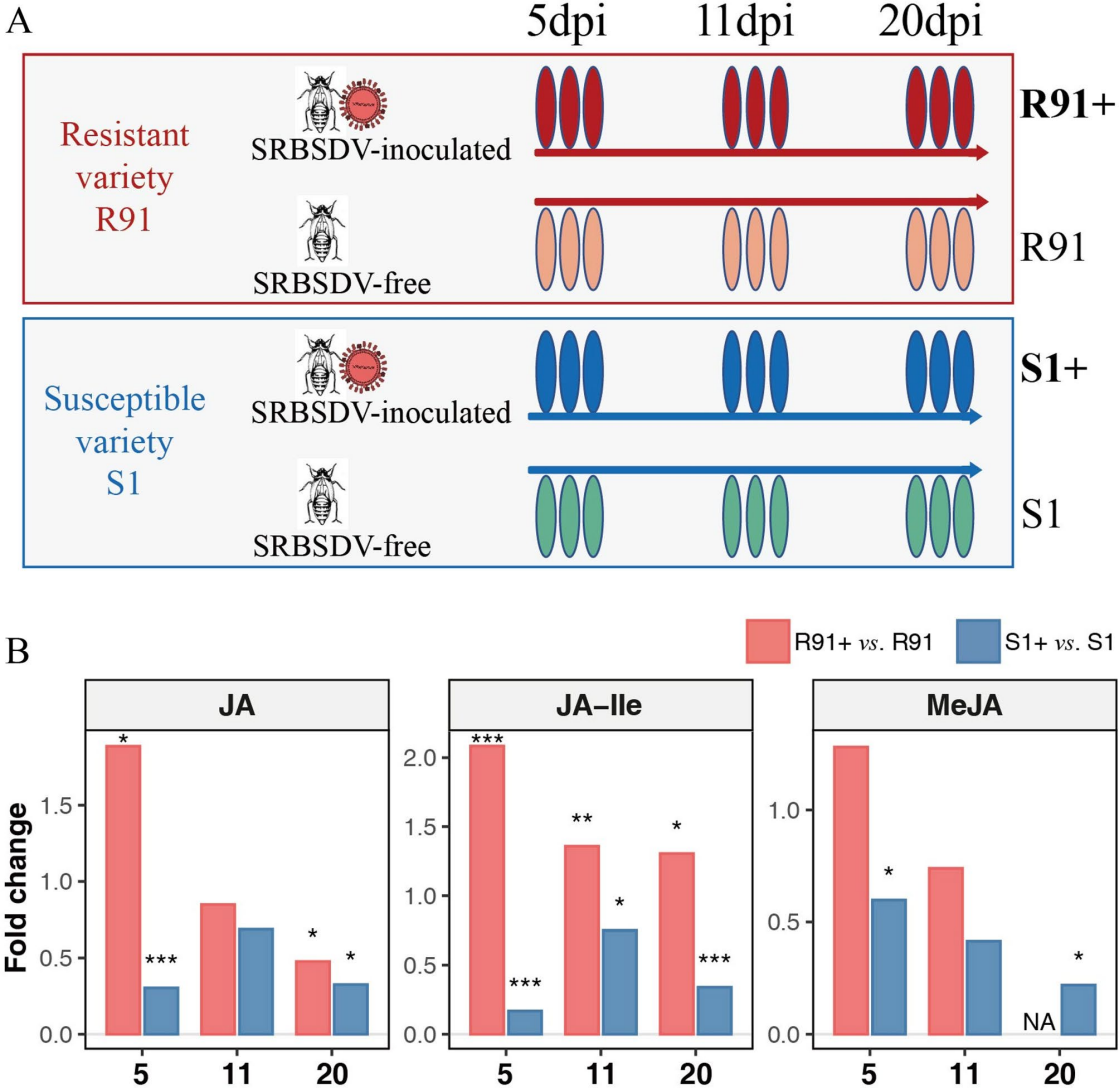
R91 exhibits rapid JA biosynthesis upon SRBSDV inoculation. (**A**) schematic representation of the experimental setup used in this study. The figure illustrates the timeline of SRBSDV infection in resistant (R91) and susceptible (S1) rice cultivars, with sampling points indicated at 5, 11, and 20 dpi. “R91+” and “S1+” represent rice individuals that were treated by SRBSDV-inoculated white-backed planthopper (WBPH), and “R91” and “S1” represent the rice individuals were treated by SRBSDV-free WBPH. (**B**) Temporal dynamics of JA, JA-Ile, and MeJA accumulation (fold change: inoculated plants compared to mock plants) in R91 and S1. Statistical significance was determined by a two-tailed Student’s *t*-test with three biological replicates (*p < 0.05, **p < 0.01, ***p < 0.001)

To elucidate the hormonal dynamics associated with SRBSDV resistance, we systematically quantified major phytohormones including GA, ABA, Auxin, Cytokinin, BRs, JAs, and SA both in R91 and S1 (one of the most susceptible accessions in the panel) at 5-, 11-, and 20-days post-inoculation (dpi) with SRBSDV (Fig. 2A and Table S5). Rice plants infected with virus-free WBPH served as mock controls. Strikingly, JA, MeJA, and JA-Ile exhibited contrasting profiles between the two accessions after inoculation. In R91, JA levels surged by 1.88-fold, while JA-Ile increased by 2.08-fold at 5 dpi compared to mock-treated controls (Fig. 2B and Table S6). Conversely, the susceptible accession S1 showed a 70% reduction in JA and an 84% reduction in JA-Ile compared to mock at 5 dpi (Fig. 2B). Similarly, MeJA levels in R91 increased by 1.28-fold at 5 dpi, while S1 experienced a 40% decline (Fig. 2B). JA induction in R91 was transient, halting by 11 dpi. However, JA-Ile remained elevated at both 11 dpi (1.36-fold) and 20 dpi (1.31-fold), while S1 maintained suppressed JA-Ile levels at all time points (11 dpi: 25% decrease; 20 dpi: 66% decrease).

### R91 exhibits early and robust gene activation in response to SRBSDV

To further elucidate the molecular mechanisms of SRBSDV resistance in rice, we conducted a comprehensive time-series transcriptome analysis using the same materials as in the hormone assay.

**Fig. 3:**
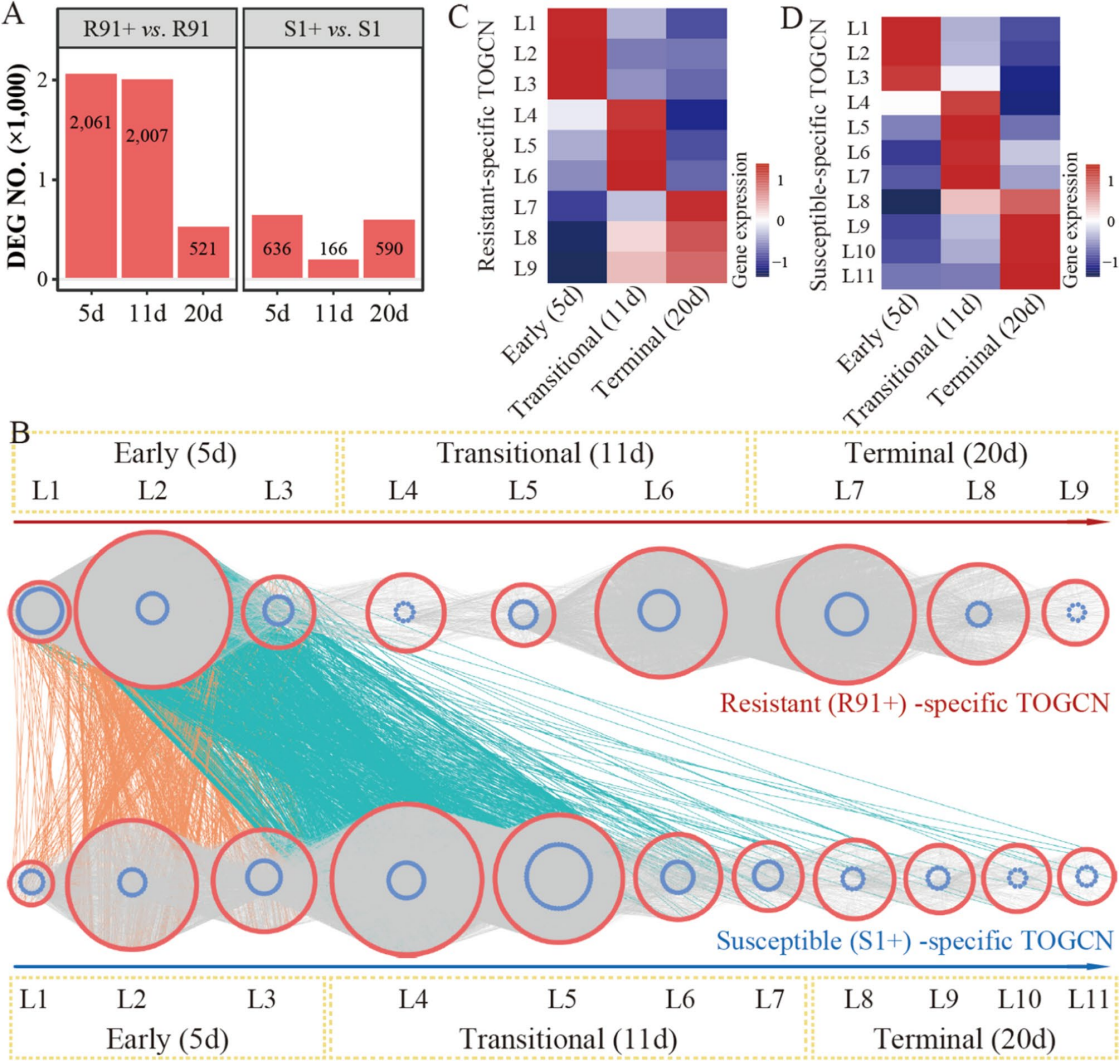
Gene expression profile and TOGCN analysis in R91 and S1. (**A**) The number of differentially expressed genes (DEGs) identified between SRBSDV-infection group and virus-free group in resistant (R91) and susceptible (S1) accessions at different time points (5, 11, and 20 days) post-inoculation. (**B**) Predicted regulatory network and the connections among TFs and structural genes. Inside the blue circles, the nodes represent TF genes. Outside the red circles, the points represent the structural genes. L1 to L11 indicate the levels identified in the time-ordered gene co-expression network (TOGCN). The resistant-specific TOGCN is shown above, while the susceptible-specific TOGCN is below. Within the same TOGCN, gray lines indicate co-expression relationships between genes. Between the two TOGCNs, orange lines connect genes that are present in the early sub-networks of both TOGCNs, while blue lines link genes that are located in the early sub-network of the resistant-specific TOGCN with those in the transitional sub-network of the susceptible-specific TOGCN. **C** and **D** show the expression profiles of the resistant-specific TOGCN and susceptible-specific TOGCN, respectively. The heatmaps of average normalized TPMs (z-score) at each stage and level identified in TOGCNs. Three stages were identified as the early (5 dpi), transitional (11 dpi) and terminal (20 dpi) stages, based on the expression profile. The bar represents the expression level of each gene (z-score). Low to high expression is indicated by a change in color from blue to red

R91 exhibited an early and robust gene activation response to SRBSDV. This was evident from the rapid and extensive transcriptional response observed as early as 5 dpi, with over 2,000 differentially expressed genes (DEGs) detected at both 5 dpi and 11 dpi (Fig. 3A). In contrast, the S1 displayed a muted response, with only a few hundred DEGs identified at 5 dpi and 20 dpi, notably only 166 DEGs at 11 dpi (Fig. 3A). Further analysis of the DEGs revealed that R91 exhibited 1,804 and 1,908 up-regulated genes at 5 dpi and 11 dpi, respectively, while S1 showed only 244 upregulated genes out of 636 DEGs at 5 dpi (Fig. 3A and Table S7). This disparity highlights the distinct capabilities of the two accessions in mounting a defense response, with R91 showing a more dynamic and extensive reaction.

### Temporal gene co-expression patterns distinguish resistance and susceptibility responses to virus inoculation

We utilized time-series transcriptome data to construct comprehensive TOGCNs, from which we unveiled distinct patterns within the (R91+) resistant-specific network and the (S1+) susceptible-specific network (Fig. 3B). The resistant-specific TOGCN consisted of 44,087 edges, while the susceptible-specific TOGCN comprised 33,884 edges (Table S8). These networks highlighted co-expression interactions among 4,167 genes (including 291 transcription factors, TFs) in the resistant-specific TOGCN and 3,676 genes (including 294 TFs) in the susceptible-specific TOGCN (Table S8). The resistant-specific network was structured into nine time-ordered gene co-expression levels (L1–L9), whereas the susceptible-specific network was segmented into eleven levels (L1–L11) (Fig. 3B). Notably, in the resistant-specific TOGCN, levels L2, L6, and L7 exhibited the highest number of edges and gene nodes, while in the susceptible-specific TOGCN, levels L4, L5, and L2 displayed the highest connectivity (Fig. 3B and Table S8).

Based on differential expression profiles, these levels were further categorized into three temporal sub-networks: the early sub-network (corresponding to 5 dpi), the transitional sub-network (11 dpi), and the terminal sub-network (20 dpi) (Fig. 3C, D). Within the resistant-specific TOGCN, the early sub-network (encompassing levels L1–L3) was notably enriched, housing more than 1,980 genes and 123 TFs compared to the other sub-networks (Table S8). This early sub-network also displayed a higher number of edges (23,673) and a greater average degree (47.82), indicating a robust transcriptional response at the gene level (Table S8).

Conversely, the transitional sub-network of the susceptible-specific TOGCN contained more genes (1,917) and TFs (163) compared to the other sub-networks and also exhibited more edges (25,095) and a higher average degree (52.36) (Table S8). Intriguingly, genes that were responsive in the early sub-network of the resistant-specific TOGCN were found to be activated during the transitional stage in the susceptible-specific TOGCN, suggesting a potential overlap or shared response mechanisms between the two networks (Fig. 3B).

### Transcriptome analysis revealed early rapid activation of JA biosynthesis genes

**Fig. 4:**
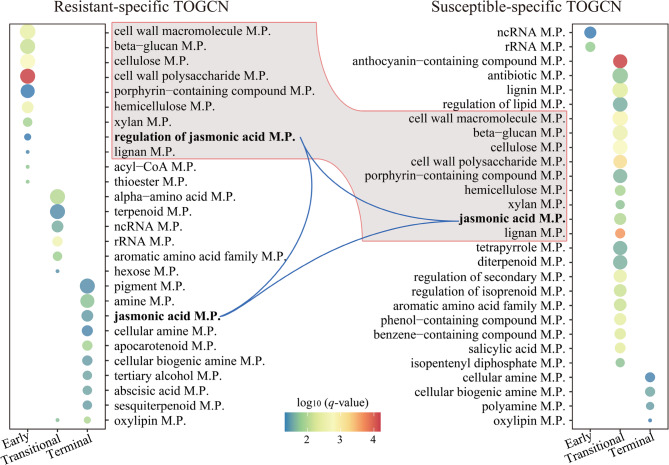
Overrepresented GO terms for co-expressed genes at each sub-network in resistant- and susceptible-specific TOGCN. The enriched metabolic pathways (M.P.) with *q*-value < 0.01 are presented. The color of circles represents the statistical significance of enriched GO terms. The size of the circles represents the number of genes in a GO term

To reveal significant differences in categories and temporal patterns of genes between resistant and susceptible accessions, Gene Ontology (GO) enrichment for co-expressed genes within the resistant-specific and susceptible-specific TOGCNs was analyzed (Fig. 4). In the resistant-specific TOGCN, key pathways were consistently enriched across all three stages. In the early sub-network, metabolic pathways related to hemicellulose, cell wall macromolecules, and polysaccharides were overrepresented (Fig. 4). The transitional sub-network showed enrichment in pathways associated with terpenoids, alpha- and aromatic amino acids, while the terminal sub-network exhibited enrichment in pathways linked to pigments, JA, and ABA. Conversely, the susceptible-specific TOGCN displayed most functional pathways enriched in the transitional stage sub-network, including noncoding RNA, anthocyanins, plant hormones, cell wall components, and diterpenoids, typically associated with disease susceptibility. Interestingly, pathways enriched in the early sub-network of the resistant-specific TOGCN overlapped with those enriched in the transitional sub-network of the susceptible-specific TOGCN, with a notable emphasis on JA related pathways, a critical factor in SRBSDV resistance (Fig. 4).

**Fig. 5:**
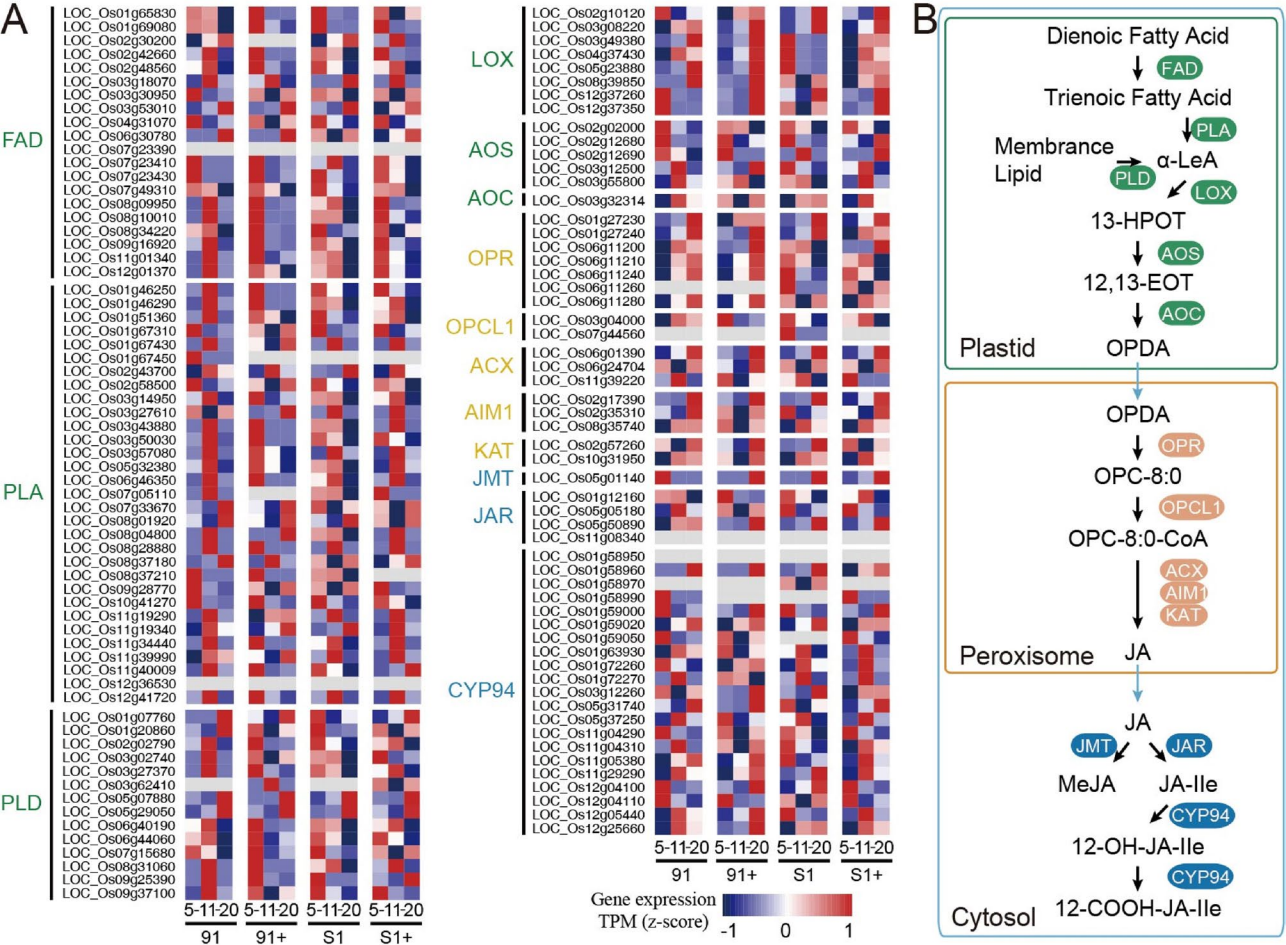
JA biosynthesis pathways and gene expression profiles. (**A**) Heatmap illustrating the expression levels of key genes involved in the JA biosynthesis pathway in both resistant (R91 + and R91) and susceptible (S1 + and S1) cultivars at three time points post-inoculation. Gene expression profiles (in normalized TPMs) in different time points (here 5, 11, 20 dpi, from left to right in each heatmap panel) are presented in the heatmap alongside the gene names. The bar represents the expression level of each gene (z-score). Low to high expression is indicated by a change in color from blue to red. FAD: Fatty acid desaturases; PLA: Phospholipases A; PLD: Phospholipases D; LOX: Lipoxygenases; AOS: Allene oxide synthases; AOC: Allene oxide cyclases; OPR: OPDA reductases; OPCL1: OPC-8:0 CoA ligase 1; ACX: Acyl-CoA oxidase; AIM1: Abnormal inflorescence meristem1; KAT: Ketoacyl-CoA-thiolases; JMT: Jasmonic acid carboxyl methyltransferase; JAR: JA-amino acid synthetase; CYP94: Cytochrome P450 monooxygenase subfamily CYP94. (**B**) Working model of JA biosynthesis and metabolic pathway

The transcriptome analysis revealed rapid activation of JA-related pathway genes, aligning with the metabolic analysis that showed significantly higher JA levels in R91 compared to S1. To further elucidate the JA pathway’s role in regulating rice resistance to SRBSDV, we reconstructed the JA biosynthesis and metabolic pathway in rice, in which 121 genes encoding 14 enzymes involved in JA biosynthesis were identified (Fig. 5, Table S3 and Table S10). The gene family encoding phospholipases A (PLA) was the largest, with 31 members, while families for allene oxide cyclases (AOC) and jasmonic acid carboxyl methyltransferase (JMT) each had one member (Table S9). Smaller families such as OPC-8:0 CoA ligase 1 (OPCL1) and ketoacyl-CoA-thiolases (KAT) contained only two members each, playing crucial roles in JA biosynthesis (Fig. 5 and Table S3).

Analysis of expression pattern of JA-related genes revealed that, at 5 days post-inoculation (dpi), 44.63% of JA-related genes were upregulated in virus-infected resistant plants (R91+) relative to their control, while only 24.79% of JA-related genes in uninfected R91 controls showed higher expression than inoculated plants. In susceptible plants (S1+), a weaker induction was observed, with 29.75% of JA-related genes upregulated upon infection compared to their controls. In the S1 mock controls, 41.32% of JA genes displayed higher expression than infected plants (Table S11).

Fast-response genes, highly expressed in R91 + during the early stage and in S1 + during the terminal stage (20 dpi), were identified. Notably, no fast-response genes were found in critical families AOC, JMT, and KAT (Table S9). However, larger families exhibited significant proportions of fast-response genes, with 75% of the FAD family, 71.42% of the PLD family, and 48.38% of the PLA family classified as fast responders (Table S9).

We further analyzed the dynamic expression patterns of genes involved in JA signaling pathway, including the *COI* (3 members) and *JAZ* (16 members) families as well as *OsMYC2* (Fig. S2 A–C and Table S12). The results uncovered distinct temporal expression dynamics in response to SRBSDV inoculation: while most of JA signaling-related genes in resistant R91 plants sustained elevated expression until the terminal stage (20 dpi), genes in the susceptible S1 exhibited significant upregulation at the transitional stage (11 dpi) (Fig. S2).

### Hierarchical regulation of JA biosynthesis associated with disease resistance in R91

**Fig. 6:**
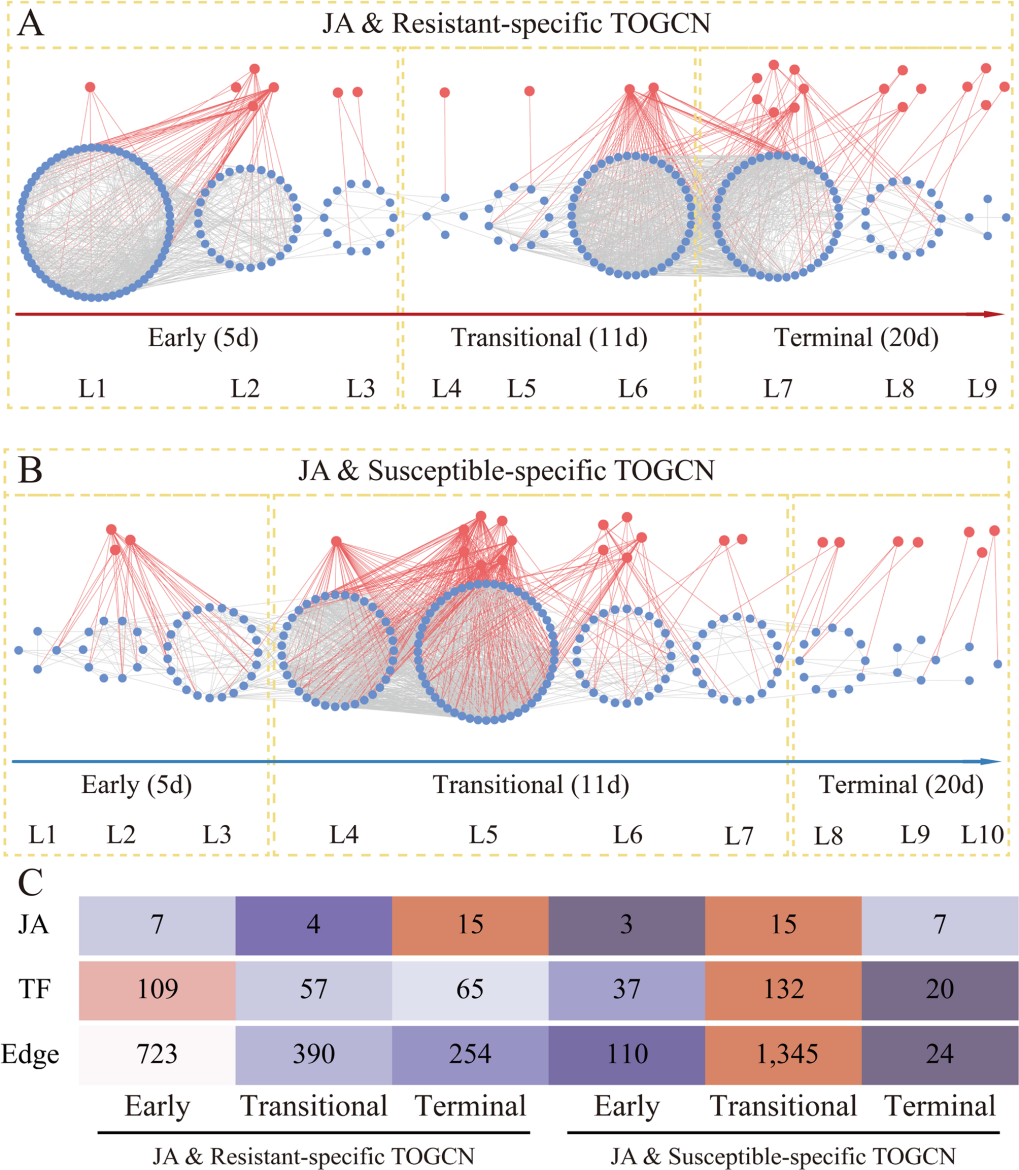
Predicted TOGCNs among TFs and enzymatic genes involved in JA biosynthetic pathways for R91 and S1. (**A**) JA & Resistant-specific TOGCN. (**B**) JA & Susceptible-specific TOGCN. (**C**) Numbers of TFs and enzymatic genes involved in JA metabolic pathways at three stages (the early, transitional, and terminal stages) in the two TOGCNs. Red nodes represent JA genes, and blue nodes represent TFs. Edges represent co-expression relationships. Edges were not shown between enzymatic genes.

To further characterize key module regulating rice SRBSDV resistance by JA, we reconstructed TOGCNs related to JA biosynthesis for both R91 and S1 after SRBSDV inoculation (Fig. 6A, B). In the JA & resistant-specific TOGCN, both the early and terminal sub-networks exhibited a significantly higher number of genes and edges, particularly involving transcription factors (Fig. 6A). The early sub-network contained 114 genes and 716 edges, with a notable presence of 109 TFs, indicating a robust and rapid initial response to JA biosynthesis (Fig. 6A and Table S13). Additionally, the terminal sub-network showed substantial activity with 80 genes, 268 edges, and 65 TFs, suggesting sustained regulatory activity in the later stages of the response (Fig. 6A and Table S13).

Conversely, the JA and susceptible-specific TOGCN displayed a different pattern, with the transitional sub-network showing the highest concentration of genes and edges (Fig. 6C and Table S13). This sub-network comprised 147 genes (including 15 JA genes and 132 TFs), and 1345 edges, suggesting a delayed but intense response during the transitional stage (Fig. 6C and Table S13). The early and terminal sub-networks in the susceptible line were relatively less active, with the early sub-network containing only 40 genes and 110 edges (including 37 TFs), and the terminal sub-network having 27 genes and 24 edges (with 20 TFs) (Fig. 6C and Table S13).

### Key module of JA signal pathway and a candidate functional gene related to SRBSDV resistance

**Fig. 7:**
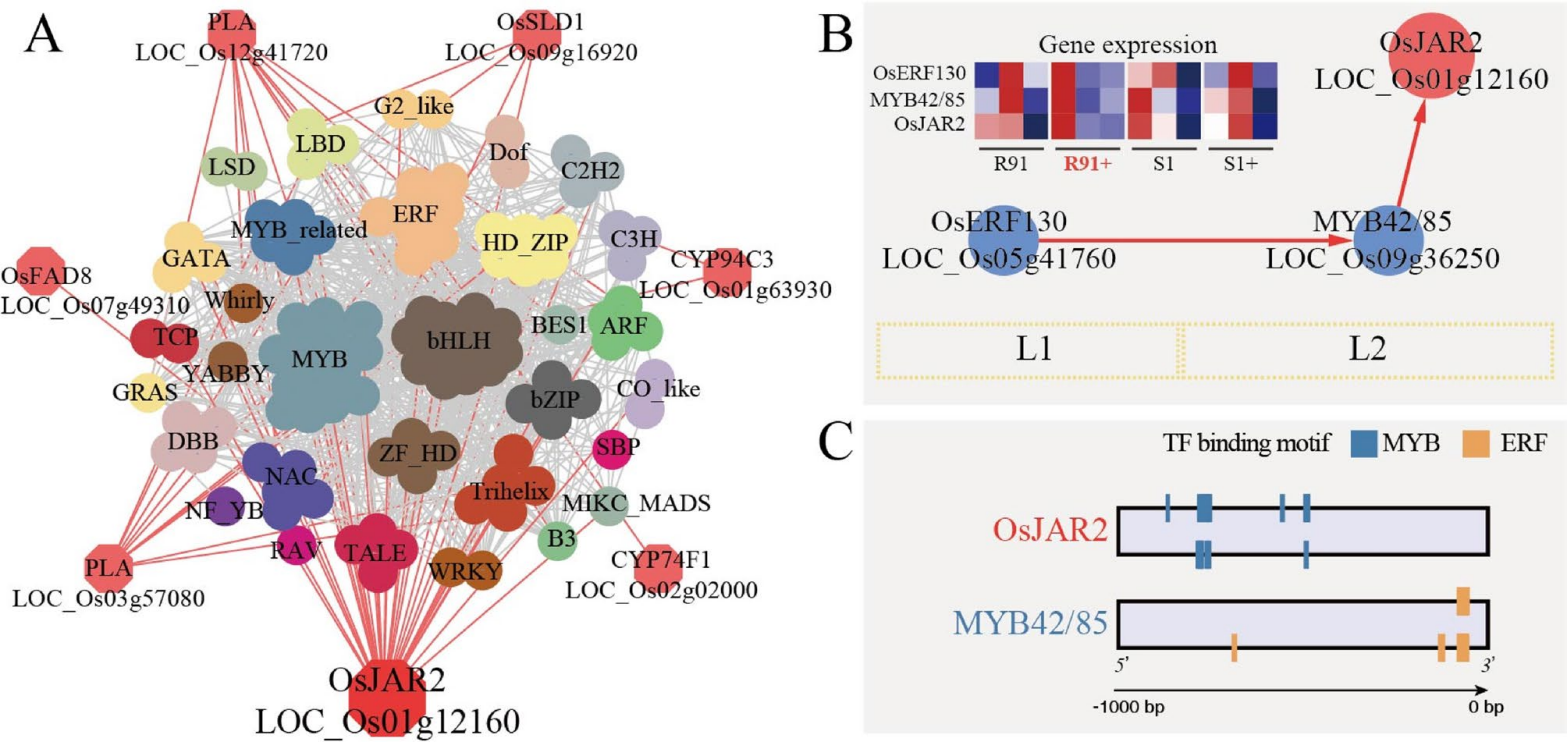
Identification of key regulatory modules in the JA pathway. (**A**) The early sub-network extracted from the resistant-specific TOGCN focusing on the JA biosynthesis pathway. (**B**) Hierarchical regulatory network resolved for the hub gene *OsJAR2*. The heatmap in the top-left corner displays gene expression profiles (normalized TPMs) at 5, 11, and 20 dpi (from left to right in each panel) for R91, R91+, S1, and S1+. Expression levels range from low (blue) to high (red). (**C**) TF binding site (TFBS) detected in the 1 Kb upstream sequences of the hub gene and the potential regulator (here, TFs; *MYB42/85*)

Focusing on the early sub-network of the JA & resistant (R91+)-specific TOGCN, we identified a key module potentially critical for the rapid biosynthesis of defense compounds mediated by JA in R91 (Fig. 7A). For JA biosynthesis, we predicted seven enzymatic genes that were expressed at high levels and directly regulated by 109 potential regulators, predominantly from the MYB (14 members), the basic helix–loop–helix (bHLHs, 12 members), and ethylene-responsive element binding factors (ERFs; 8 members) families (Fig. 7A and Table S14). In gene co-expression networks, nodes with high centrality typically represent core factors essential to network function. Within this module, JA-amino acid synthetase 2 (*OsJAR2*; *LOC_Os01g12160*) was identified as a hub gene exhibiting the highest centricity degree (34) among other JA related genes (Fig. 7A and Table S15). *OsJAR2* is a crucial rate-limiting enzyme in the JA biosynthesis pathway, which catalyzes the synthesis of JA-Ile, the bioactive form of JA. Consistent with this observation, SRBSDV infection induced contrasting JA-Ile dynamics - rapid and sustained accumulation in the resistant genotype R91 versus progressive depletion in the susceptible S1 (Fig. 2B).

Within the early sub-network of the JA & resistant (R91+)-specific TOGCN, two transcription factors, *MYB42/85* (*LOC_Os09g36250*; Degree = 816) and *OsERF130* (*LOC_Os05g41760*; Degree = 795), exhibit exceptional connectivity within the same sub-network. In this hierarchical network, *OsERF130* occupies the top tier (level L1), while *MYB42/85* and *OsJAR2* reside at the subsequent level (L2) (Fig. 7B). There was also a strong gene co-expression among the three genes in R91+ (Fig. 7B). Furthermore, the correlation between *OsJAR2* and *MYB42/85* is exceptionally high (*r* = 0.96), as was that between *OsERF130* and *MYB42/85* (*r* = 0.95) (Table S16). Complementing these findings, TF DNA binding site prediction analysis revealed putative ERF motifs within 1 kb upstream of *MYB42/85*’s transcription start site and MYB motifs within 1 kb upstream of *OsJAR2* (Fig. 7C). Together, these data support a hierarchical regulatory cascade wherein *OsERF130* potentially functions as an upstream regulator of *MYB42/85*, which directly modulates *OsJAR2* expression.

### GWAS and haplotype analyses implied *OsJAR2* as a key regulator of SRBSDV resistance in rice

**Fig. 8:**
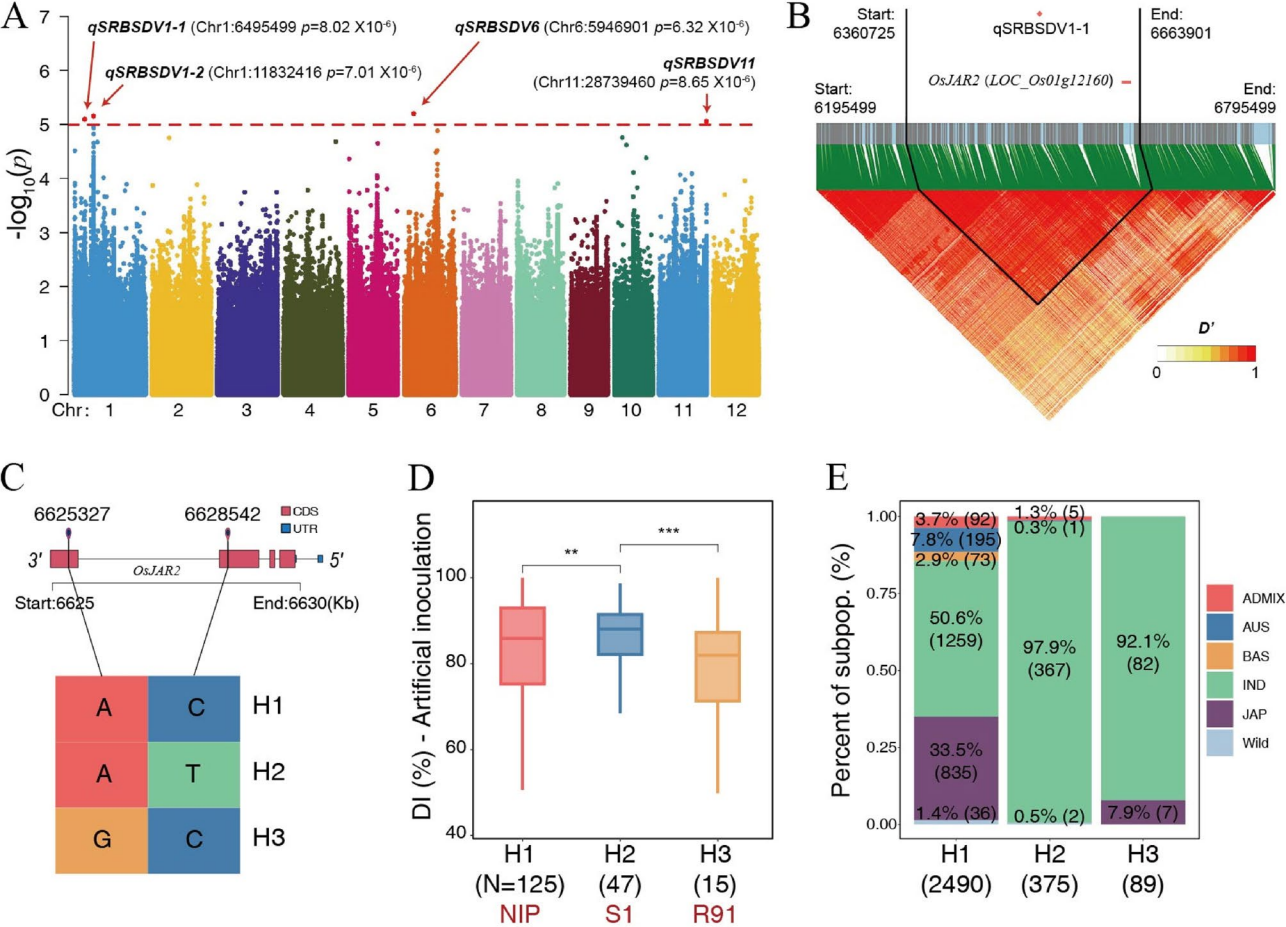
GWAS analysis of SRBSDV resistance in rice. (**A**) QTLs associated with SRBSDV resistance identified by GWAS. (**B**) *qSRBSDV1-1* locus on chromosome 1, spanning approximately 300 kb from 6,360,725 bp to 6,663,901 bp. Heatmap showing local linkage disequilibrium (LD) patterns around *qSRBSDV1-1* locus. (**C**) Haplotypes of *OsJAR2*. (**D**) SRBSDV disease incidence of different haplotypes among 195 accessions. Statistical significance was determined by a two-tailed Student’s t-test, indicated by asterisks (***p < 0.001; **p < 0.01), calculated using Student’s *t*-test. (**E**) The frequency distributions of three haplotypes in 3K rice genomes

To further validate the genetic association between *OsJAR2* and resistance to SRBSDV, we conducted GWAS analysis using the DI as phenotype, and resequencing data of the 195 accessions. The GWAS results demonstrated 4 novel SRBSDV resistance quantitative trait loci (QTLs) in chromosome 1, 6, and 11 (Fig. 8A; Table 1). Based on the analysis of local linkage disequilibrium (LD) patterns, we delineated the *qSRBSDV1-1* locus on chromosome 1, spanning approximately 300 kb from 6,360,725 bp to 6,663,901 bp (Fig. 8B). Within this interval, 46 protein-coding genes were annotated (Table S17). Differential expression analysis across multiple time points identified only four genes showing significant expression differences between virus-inoculated and control groups (Fig. S3 and Table S17). Among these, two genes—*LOC_Os01g12000* and *LOC_Os01g12160*—exhibited consistent differential expression in both the susceptible line S1 and the resistant line R91. Notably, *LOC_Os01g12000* is annotated simply as an “expressed protein” with no known function, whereas *LOC_Os01g12160* (*OsJAR2*) was identified as putative hub gene regulating SRBSDV resistance via the JA pathway in this study. Further TOGCNs analysis revealed that only five genes were unique to either the resistant (R91+)-specific or susceptible (S1+)-specific TOGCNs. Among these, *OsJAR2* was the sole gene whose network position differed between the two TOGCNs (Table S17). Integrating all these evidences, we propose that OsJAR2 is likely to be the functional gene underlying *qSRBSDV1-1*.

We further explored the genetic basis of *OsJAR2* in conferring SRBSDV resistance by haplotype analysis. Genome-wide resequencing of the 195 accessions identified three non-synonymous single nucleotide polymorphisms (nsSNPs) in *OsJAR2*, leading to the characterization of three haplotypes (H1, H2, and H3) (Fig. 8C and Table S18). In both our sequenced germplasm collection and the 3K rice genomes (sequence data from https://iric.irri.org/resources/snp-seek-datasets), haplotype H1 was the most prevalent, followed by H2, with H3 being the least frequent (Fig. 8D, E). Nipponbare (NIP) belongs to haplotype H1, the susceptible line S1 corresponds to H2, and the resistant line R91 falls into H3 (Fig. 8D).

Association analysis demonstrated significant phenotypic divergence in DI among the haplotypes. Accessions with the H3 haplotype exhibited the lowest DI (*p*-value < 0.001) (Fig. 8D). Compared to H1, H3 differed by only a single nsSNP at position Chr1:6,628,542 (Fig. S4A). This nsSNP resulted in a valine-to-alanine substitution at amino acid residue 471, which is located within the interior of the C-terminal domain of the OsJAR2 protein (Fig. S4B).

The H3 haplotype exhibited large protective effects, reducing disease incidence by 13.33% (Cohen’s d = − 1.23, *P* < 0.001) compared to the susceptible H2 haplotype and 9.73% (d = − 0.64, *P* < 0.05) relative to the prevalent H1 haplotype. This genetic stratification highlights *OsJAR2* as a central regulator of JA-mediated antiviral defense and identifies H3-associated SNPs as valuable molecular markers for breeding SRBSDV-resistant rice varieties. H1 encompassed the greatest genetic diversity and represents the ancestral haplotype, whereas haplotype H3 consisted entirely of cultivated rice varieties (Fig. 8E). These observations suggest that H3, as a rare haplotype, most likely arose during the selective breeding of cultivated rice. Further geographic analysis revealed that accessions carrying H3 originated from China, India, Sri Lanka, Vietnam, and Senegal (Table S18), highlighting these regions as potential sources of resistance for targeted breeding programs.

## Discussion

### Discovery of R91 as a disease-resistant germplasm and its dual-resistance breeding potential

SRBSDV poses a devastating threat to rice production, yet the scarcity of highly resistant germplasms has severely hindered disease-resistance breeding. Through systematic screening of 195 rice accessions spanning 34 countries and 13 geographic regions [12], we identified R91, originating from India, exhibiting stable high resistance to SRBSDV in both field trials and artificial inoculations (Fig. 1A, D). This remarkable resistance outperforms most reported resistant resources, positioning R91 as a globally rare SRBSDV-resistant germplasm. Notably, our previous study has already identified R91 as a robust resistance germplasm against RBSDV [7]. Such dual resistance provides a foundational resource for breeding programs targeting overlapping viral epidemics, simplifying multi-resistance cultivar development while mitigating risks associated with viral evolution in the field.

Further analysis of resistance distribution revealed that varieties from South Asia Central (SAC; India, Bangladesh, Nepal) exhibited significantly higher resistance, contrasting with East Asian accessions (China, Japan) that displayed higher susceptibility. This geographic pattern aligns with the migration route of the white-backed planthopper, the SRBSDV vector, across South and Southeast Asia [26], as well as historical prevalence of viral diseases in these regions [4]. Strikingly, 15 out of the top 50 most resistant accessions originated from South Asia (Fig. 1C), suggesting long-term natural selection pressure may have enriched resistance alleles in these germplasms. For example, R91’s resistance likely evolved through co-adaptation with indigenous WBPH populations and viral strains, leading to fixation of advantageous alleles via gene-environment or gene-gene interactions.

The dual resistance of R91 to both SRBSDV and RBSDV implies potential overlap in defense mechanisms against these phylogenetically related Fijiviruses. Our previous work identified the aspartic protease gene *OsAP47* as a key player in RBSDV resistance, conferring protection via degradation of viral capsid proteins [7]. Given the structural and replicative similarities between SRBSDV and RBSDV [5, 27], we hypothesize that OsAP47 may target conserved viral domains to achieve cross-resistance. Furthermore, our study revealed rapid activation of the JA pathway in R91 during early SRBSDV infection (Figs. 2B and 5A). Interestingly, JA-Ile has been reported to induce after RBSDV infection [28], which implies its critical role as a common regulatory node coordinating dual resistance to both SRBSDV and RBSDV.

The dual resistance of R91 against both RBSDV and SRBSDV establishes it as an invaluable genetic asset, laying the groundwork for the development of rice varieties with improved resistance profiles. Its robust resistance traits offer a foundation upon which to dissect the complex dynamics of rice-virus interactions and to expedite the incorporation of resistance into commercially viable rice cultivars. Consequently, the use of R91 in molecular breeding has the potential to significantly fortify rice against these two viruses, providing a boon to global food security by mitigating virus-induced yield losses.

### Temporal hierarchies of transcriptional activation and JA pathway in early defense are critical for the formation of SRBSDV resistance

In this study, we aimed to explore the molecular mechanisms underlying rice resistance to SRBSDV by leveraging multi-omics approaches, which allow us to analyze the comprehensive omics landscape and the molecular responses associated with plant defense [29]. We specifically examined the transcriptional dynamics of the resistant accession, R91, in comparison to a susceptible accession, S1, as well as conducted a metabolomics analysis of plant hormones after SRBSDV inoculation.

The results revealed that R91 mounts an immediate and robust defense response, as evidenced by the rapid upregulation of defense-related genes from as early as 5 dpi. Conversely, S1 manifested a lagged transcriptional response. This marked difference underscores R91’s enhanced ability to rapidly engage defenses upon infection, underscoring the critical role of early gene activation in establishing SRBSDV resistance in rice. Similar to our findings, previous research has indicated that upon virus inoculation, resistant ecotypes can trigger an extensive and rapid transcriptomic remodeling, including genes involved in plant-pathogen interaction, plant hormone signal transduction, the MAPK signaling pathway or ubiquitin mediated proteolysis [30]. In this study, we identified a distinctly earlier transcriptomic response between the resistant and susceptible accession, further emphasizing the significance of early and substantial transcriptomic remodeling in establishing SRBSDV resistance.

Further TOGCNs analysis illuminated the distinct temporal gene expression patterns between the resistant and susceptible accessions. The TOGCN specific to R91 exhibited enhanced connectivity and gene interactions, particularly involving key transcription factors, suggesting a sustained and comprehensive transcriptional response. In contrast, S1’s co-expression network showed a resurgence of gene activity primarily during the transitional phase, which signifies a postponed transcriptomic response to the virus.

Delving deeper into transcriptomic data reveals that the JA pathway is significantly activated in R91 during the early stages of infection. A total of 44.63% of JA-related genes were upregulated in R91, in contrast to only 29.75% in S1. This pronounced activation underscores that the rapid induction of JA pathway is critical for SRBSDV resistance. Notably, metabolomics analysis confirmed that JA, JA-Ile and MeJA were significantly more induced in R91 than in S1 in early stage of virus infection. Although the increases in JA and JA-Ile appear moderate, we observed a coordinated elevation of JA, JA-Ile, and MeJA in resistant plants following infection, whereas these hormones declined significantly in susceptible accessions. This contrasting pattern strongly supports the critical role of JAs in SRBSDV resistance. Moreover, the relatively modest induction of JA may also stem from tissue-specific accumulation, temporal variation in sampling, or rapid metabolic conversion into downstream derivatives-aspects that warrant further investigation to fully elucidate.

It has been reported that RBSDV infection can alter hormone levels, influencing the plant’s defense activation and suscepand its derivativetibility manipulation [31]. In this study, for the first time, we identified different JA synthesis gene expression, as well as JA and its derivative contents between resistant and susceptible rice.

During the co-evolution between virus and plant, plants have developed a sophisticated and multifaceted immune system that includes a complex interplay between different phytohormones [32, 33]. JAs are a class of hormones playing vital roles in plant defense responses to abiotic and biotic stresses, including viral attacks [34, 35]. Our findings align with the established role of JA in plant defense against viral infection [36, 37]. As previous studies have shown, the JA pathway plays a significant role in plant defense mechanisms, including the activation of pathogenesis-related genes and the induction of initial immune responses to viral infections [38, 39]. For example, continuous JA treatment decreased the DNA titer of beet curly top virus, indicating that suppression of the JA response may be critical for geminivirus infection [40]. Suppression of JA signaling has also been seen in rice ragged stunt virus infection [41]. Our results showing increased JA synthesis pathway and contents further highlight the importance of JA in regulating resistance to SRBSDV in rice.

Interestingly, JA also plays a crucial role as a regulator in how plants respond to insect infections [42]. Moreover, it has been reported that WBPH infestation down-regulates the expression of JA pathway genes in rice, suggesting that JA pathways can be influenced by viral infection and insect feeding [43]. However, it is noteworthy that despite the rapid and significant synthesis of JA observed in the R91, our earlier research indicated that R91 does not demonstrate resistance to insect attacks [7]. Consequently, gaining a more nuanced understanding of the relationship between the JA response and the resistance to both insect pressures and the SRBSDV warrants further exploration and attention.

### *OsJAR2*: a candidate functional gene and molecular hub bridging JA dynamics and SRBSDV resistance

Currently, genetic studies on SRBSDV resistance in rice remain limited, with no functionally validated resistance genes reported [44, 45]. In this study, we identified four novel QTLs associated with SRBSDV resistance, one of which co-localized with *OsJAR2*—encoding the key enzyme that catalyzes the biosynthesis of JA-Ile, a bioactive JA derivative essential for activating downstream defense responses [46]. TOGCN analysis unveiled *OsJAR2* as the putative central hub bridging rapid JA dynamics and SRBSDV resistance. More importantly, we observed a strong positive correlation between *OsJAR2* expression and JA-Ile accumulation in resistant genotypes following SRBSDV inoculation, contrasting sharply with the suppressed response observed in the susceptible accession. Population-wide haplotype analysis further identified a resistant-associated H3 haplotype containing two non-synonymous substitutions in *OsJAR2*. Notably, the H3 haplotype was present in four of the five most resistant accessions (Table S18), implying a significant association between H3 and elevated resistance in diverse rice varieties.

Rice harbors two JAR1-like GH3 enzymes, OsJAR1 and OsJAR2, which exhibit distinct stress-responsive expression patterns [47]. It has been reported that the activity of *OsJAR1* is necessary for effective defense against the blast fungus, while another study demonstrated that *OsJAR2* is rapidly induced upon invasion by RBSDV [28]. Integrating DNA binding site predictions with co-expression network analyses, we propose that *OsJAR2* operates within a hierarchical regulatory cascade, with *MYB42/85* serving as direct regulators and *OsERF130* acting as a secondary modulator.

Our findings suggest that the amino acid substitutions in the H3 haplotype may confer resistance, likely by modulating *OsJAR2*’s enzymatic activity or protein-protein interactions—since the substituted residue lies within the C-terminal domain’s interior. However, an alternative hypothesis involving promoter polymorphisms that alter transcriptional regulation linked to these SNPs cannot be excluded. The current data cannot definitively distinguish between these mechanisms, as both coding changes and expression differences correlate with resistance. Further molecular genetic studies will be essential to resolve this question.

More importantly, we identified the H3 haplotype associated with resistance, which can serve as a practical and valuable marker for precision breeding strategies aimed at enhancing SRBSDV resistance in rice.

## Conclusions

In conclusion, this study identifies R91 as a novel and highly resistant germplasm against SRBSDV and RBSDV, providing dual resistance resources for breeding programs targeting these devastating rice viruses. The early elevation of JAs levels, as well as rapid activation of defense genes and JA biosynthesis pathway in R91 highlights the pivotal role of timely JA-mediated responses in conferring SRBSDV resistance. Multi-omics and genetic analysis identified *OsJAR2* as a potential hub gene, offering a molecular blueprint for developing rice varieties with enhanced SRBSDV resistance. These findings deepen our understanding of the molecular mechanisms underlying SRBSDV resistance and provide a foundation for innovative strategies to improve rice viral resilience, ultimately contributing to increased yield and global food security.

## Supplementary Information

Supplementary Material 1: Table S1: Disease incidence of 195 rice accessions in field trial and artificial inoculation. Table S2: The statistics for RNA sequencing data. Table S3: Known genes involved in Jasmonic acid (JA) biosynthesis pathway in rice. Table S4: The seed genes for reconstructing the TOGCNs. Table S5: Metabolite concentrations in resistant (R91) and susceptible (S1) accessions at 5-, 11-, and 20-Days Post Inoculation (dpi) with SRBSDV. Table S6: Fold change of plant hormones between inoculated and mock plants of R91 and S1. Table S7: The statistics for differential gene expression analysis. Table S8: The statistics for resistant- and susceptible-specific TOGCNs. Table S9: Distribution and rapid response genes in JA biosynthesis pathway in rice. Table S10: Expression profiles of genes involved in JA biosynthesis and metabolic pathways. Table S11: Statistics of JA genes that are upregulated at three different stages under two comparisons: R91+ vs. R91 and S1+ vs. S1. Table S12: Gene expression profiles in JA signaling pathways Table S13: Distribution of edges, genes, TF, and JA enzymatic genes across different sub-networks of resistant (R91+)-specific and susceptible (S1+)-specific TOGCNs. Table S14: Statistics of TFs clustered into the early sub-network of the JA & resistant (R91+)-specific TOGCN. Table S15: Information of genes clustered into the early sub-network of the JA & resistant (R91+)-specific TOGCN. Table S16: Pearson correlation coefficient (r) for predicting the regulatory relationship between *OsJAR2*, *ERF130* and *MYB42/85* in resistant (R91+)-specific TOGCN. Table S17: Integrated characterization of candidate genes within *qSRBSDV1-1* locus. Table S18: Association of *OsJAR2* haplotypes and SRBSDV disease incidence. Table S19: Effect size of *OsJAR2* haplotypes on disease incidences.

Supplementary Material 2: Fig. S1. Population structure analysis for 195 accessions. Fig. S2. Expression profiles of genes in JA signaling pathways. Fig. S3. Differential expression levels of genes among inoculated plants and mock plants in R91 and S1. Fig. S4. The sequence structure of two non-synonymous SNPs identified within *OsJAR2*. 

## Data Availability

The dataset generated and analyzed during the current study is available in the National Genomics Data Center (NGDC), China National center for Bioinformation (https://ngdc.cncb.ac.cn/) under the BioProject accession number PRJCA034117 (https://ngdc.cncb.ac.cn/gsa/browse/CRA021647).

## Abbreviations

AOC: allene oxide cyclases
bHLH: basic helix–loop–helix
EAS: East Asia
ERFs: ethylene-responsive element binding factors
DEGs: differentially expressed genes
DI: disease incidence
Dpi: days post-infection
FC: fold change
GO: Gene Ontology
GCN: gene co-expression network
JA: jasmonic acid
JA-Ile: jasmonic acid-isoleucine
JMT: jasmonic acid carboxyl methyltransferase
KAT: ketoacyl-CoA-thiolases
MAD: median absolute deviation
MeJA: Methyl jasmonate
MP: metabolic pathways
OPCL1: OPC-8:0 CoA ligase 1
OPDA: 12-oxo-phytodienoic acid
PLA: phospholipases A
QTLs: quantitative trait locus
RBSDV: rice black-streaked dwarf virus
SAC: South Asia Central
SER: Sea Islands
SRBSDV: Southern rice black-streaked dwarf virus
TFs: transcription factors
TOGCNs: time-ordered gene co-expression networks
TPM: transcripts per Kilobase Million
WAF: West Africa
WBPH: white-backed planthopper
