# Supplementary material for: GWAS and multi-omics study reveal OsJAR2 associated jasmonate biosynthesis contributes to Southern rice black-streaked dwarf virus resistance in rice
Source: BMC Genomics. 2025 Oct 29;26:971. doi: 10.1186/s12864-025-12159-8 (PMC12574106; doi:10.1186/s12864-025-12159-8)
Supplement: Supplementary file 2 — Supplementary Material 2: Fig. S1. Population structure analysis for 195 accessions. Fig. S2. Expression profiles of genes in JA signaling pathways. Fig. S3. Differential expression levels of genes among inoculated plants and mock plants in R91 and S1. Fig. S4. The sequence structure of two non-synonymous SNPs identified within OsJAR2. [file 12864_2025_12159_MOESM2_ESM.docx]

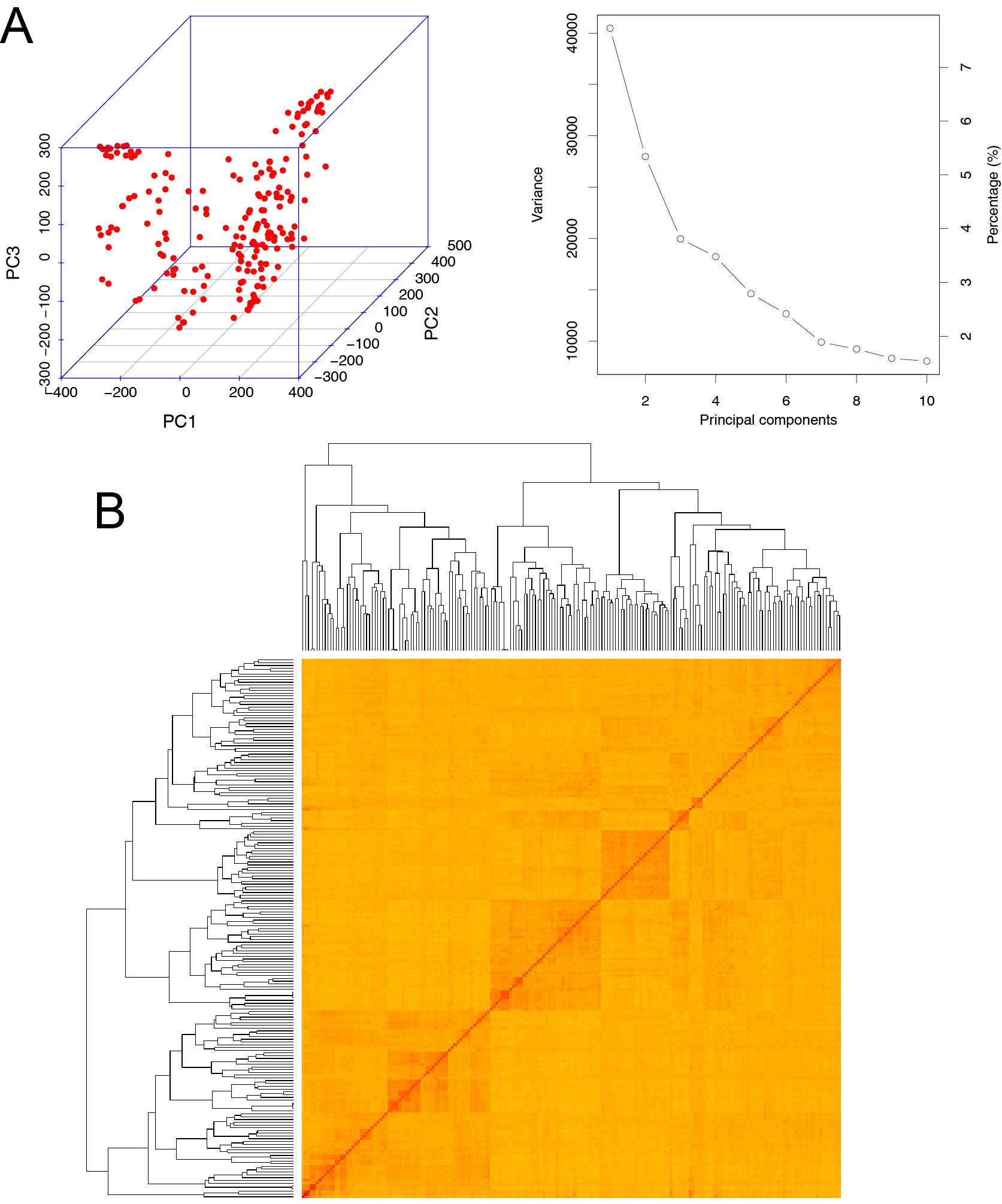


Fig. S1. Population structure analysis for 195 accessions.

A. Biplot and scree plot in principal component analysis (PCA) analysis. B. Heat map of the marker-based kinship (K) matrix using the method of VanRaden.


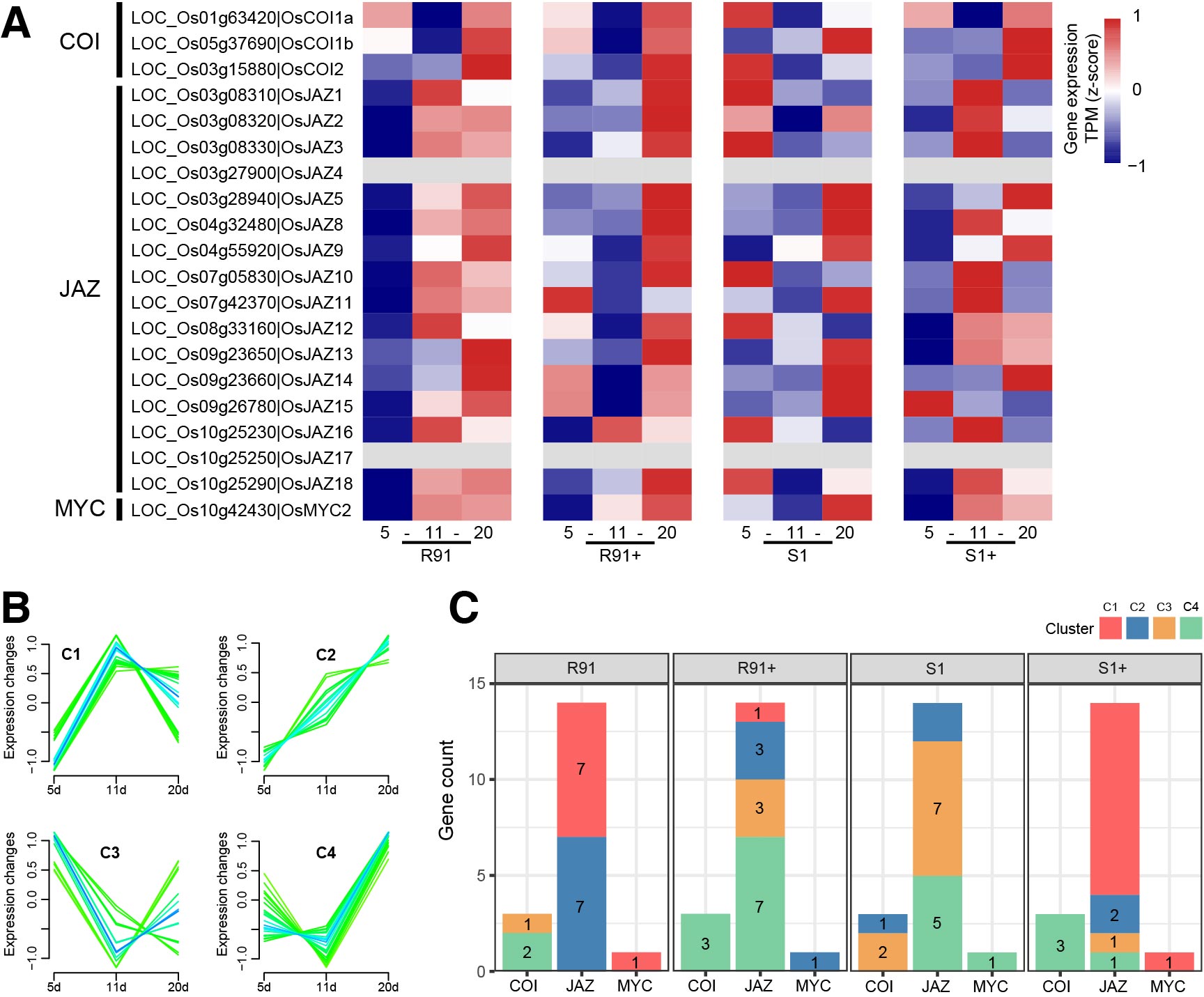


Fig. S2. Expression profiles of genes in JA signaling pathways.

A. Heatmap illustrating the expression levels of key genes involved in JA signaling pathways in both resistant (R91+ and R91) and susceptible (S1+ and S1) cultivars at various time points post-infection. COI: coronatine-insensitive; JAZ: jasmonate ZIM-domain. B. Gene co-expression clusters showing high expression in resistant (R91) and susceptible (S1) rice cultivars at different time points (5, 11, and 20 dpi). Color code reflects ‘Membership’ values calculated by Mfuzz, where magenta corresponds to high values and green to low values of Membership score. C. The numbers of genes from different clusters. Plot numbers indicate the number of genes assigned to each of these clusters and the overlap sets.


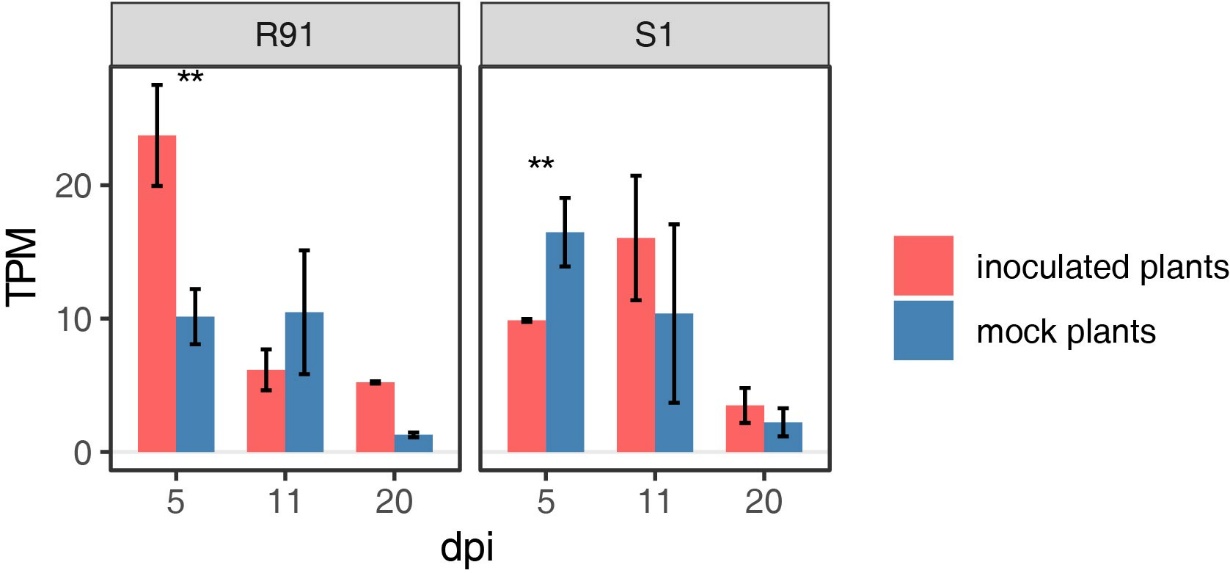


Fig. S3. Differential expression levels of genes among inoculated plants and mock plants in R91 and S1.

The adjusted *p* value calculated by DEseq2 was used to assess statistical significance (* p <0.01).


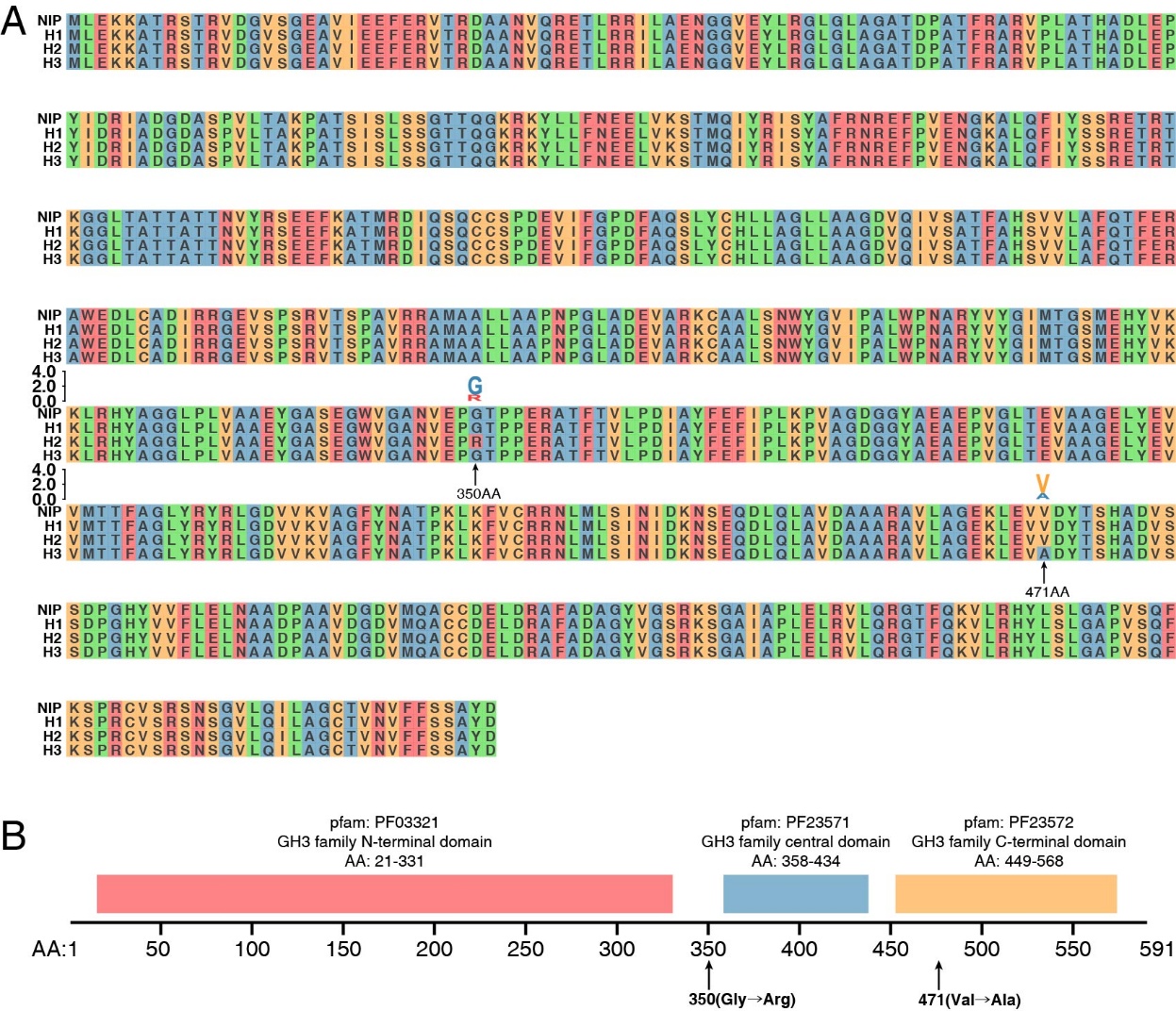


Fig. S4. The sequence structure of two non-synonymous SNPs identified within *OsJAR2.*

A. Amino acid multiple sequence alignment is between different haplotypes. B. Conserved domains of OsJAR protein. Two nonsynonymous SNPs (nsSNPs) at positions Chr1:6,625,327 and Chr1:6,628,542 correspond to amino acid residues 350 and 471, respectively. Position 471 (Val to Ala) was located within the interior of the C-terminal domain. Position 350 (Gly to Arg) was situated between the N-terminal and central domains.
